# Multiple Alternative Carbon Pathways Combine To Promote Candida albicans Stress Resistance, Immune Interactions, and Virulence

**DOI:** 10.1128/mBio.03070-19

**Published:** 2020-01-14

**Authors:** Robert B. Williams, Michael C. Lorenz

**Affiliations:** a Department of Microbiology and Molecular Genetics, University of Texas McGovern Medical School and the MD Anderson Cancer Center UTHealth Graduate School of Biomedical Sciences, Houston, Texas, USA; Duke University Medical Center

**Keywords:** *Candida*, macrophage, virulence, carboxylic acid, amino acid, *N*-acetylglucosamine, metabolism, stress, fungal pathogenesis, nutritional immunity, host-pathogen interactions

## Abstract

The phagocytic cells of the innate immune system are an essential first line of antimicrobial defense, and yet Candida albicans, one of the most problematic fungal pathogens, is capable of resisting the stresses imposed by the macrophage phagosome, eventually resulting in the destruction of the phagocyte. C. albicans rapidly adapts to the phagosome by upregulating multiple alternative carbon utilization pathways, particularly those for amino acids, carboxylic acids, and *N*-acetylglucosamine (GlcNAc). Here, we report that C. albicans recognizes these carbon sources both as crucial nutrients and as independent signals in its environment. Even in the presence of glucose, each carbon source promotes increased resistance to a unique profile of stressors; lactate promotes increased resistance to osmotic and cell wall stresses, amino acids increased resistance to oxidative and nitrosative stresses, and GlcNAc increased resistance to oxidative stress and caspofungin, while all three alternative carbon sources have been shown to induce resistance to fluconazole. Moreover, we show mutants incapable of utilizing these carbon sources, in particular, strains engineered to be defective in all three pathways, are significantly attenuated in both macrophage and mouse models, with additive effects observed as multiple carbon pathways are eliminated, suggesting that C. albicans simultaneously utilizes multiple carbon sources within the macrophage phagosome and during disseminated candidiasis. Taking the data together, we propose that, in addition to providing energy to the pathogen within host environments, alternative carbon sources serve as niche-specific priming signals that allow C. albicans to recognize microenvironments within the host and to prepare for stresses associated with that niche, thus promoting host adaptation and virulence.

## Abstract (precis)

Candida albicans is a fungal pathogen and a significant cause of morbidity and mortality, particularly in people with defects, sometimes minor ones, in innate immunity. The phagocytes of the innate immune system, particularly macrophages and neutrophils, generally restrict this organism to its normal commensal niches, but C. albicans shows a robust and multifaceted response to these cell types. Inside macrophages, a key component of this response is the activation of multiple pathways for the utilization of alternative carbon sources, particularly amino acids, carboxylic acids, and *N*-acetylglucosamine. These carbon sources are key sources of energy and biomass but also independently promote stress resistance, induce cell wall alterations, and affect C. albicans interactions with macrophages. Engineered strains incapable of utilizing these alternative carbon pathways are attenuated in infection models. These data suggest that C. albicans recognizes nutrient composition as an indicator of specific host environments and tailors its responses accordingly.

## INTRODUCTION

Candida albicans is both a common human commensal and a serious clinical problem, capable of causing an array of mucosal and invasive infections. The most severe form of infection, disseminated candidiasis, is the most common fungal nosocomial bloodstream infection (1, 2). C. albicans often infects the most vulnerable patient populations; those undergoing chemotherapy or organ transplantations or with implanted medical devices are especially vulnerable to disseminated candidiasis due to prolonged hospital stays and weakened immune systems (3). With mortality rates reaching 40% and an increasing fear of antifungal resistance, it is critical that we understand the basis of the many pathogenesis mechanisms that C. albicans utilizes during disseminated infection.

The phagocytes of the innate immune system are among the first defense mechanisms deployed to combat a *Candida* infection (4). Neutrophils and macrophages typically clear pathogens by bombarding the organism with a variety of stressors such as oxidative stress, nitrosative stress, and hydrolytic enzymes, some of which are potentiated by phagosome acidification (5–9). C. albicans, however, is capable of surviving macrophage phagocytosis by resisting these stressors, actively neutralizing the pH of the phagosome, and ultimately shifting its morphology to the hyphal form. These stresses lead to the death of the macrophage via pyroptosis and allow the fungal cell to escape the phagocyte and continue dissemination (10–17). Thus, we and others have characterized the responses that allow C. albicans to adapt and overcome the phagosomal environment. Transcriptomic studies reveal that C. albicans rapidly adapts to the macrophage phagosome by initially shifting its metabolism away from glycolysis in favor of alternative carbon sources such as carboxylic acids, amino acids, peptides, *N*-acetylglucosamine (GlcNAc), and fatty acids (10, 18–24). These compounds are sources of energy and biomass, and mutations that impair utilization of these nutrients are attenuated in macrophage and mouse virulence models.

Beyond their role as nutrients, three of these carbon sources also directly contribute to C. albicans*-*phagocyte interactions: carboxylic acids such as lactate, amino acids, and GlcNAc. Catabolism of these alternative carbon sources results in a significant increase in extracellular pH, both *in vitro* and within the phagososome, but the physiological effects diverge from there (10, 13, 14). The presence of lactate increases C. albicans resistance to a variety of host-relevant stressors, enhances biofilm formation, and reduces recognition by macrophages (15, 25–28). These adaptations are mediated in part by alterations in the thickness and composition of the cell wall (15, 26, 27). Amino acids and GlcNAc strongly induce hyphal morphogenesis, contributing to fungal survival both within macrophages and during disseminated candidiasis (10–12). How the presence of amino acids or GlcNAc might affect stress resistance, cell wall morphology, or phagocyte recognition has not previously been addressed.

The genetic requirements for the physiological changes promoted by the different carbon sources are also distinct, which has allowed the dissection of the roles of the individual alternative carbon pathways (10, 13, 14, 26, 29, 30). *JEN1* and *JEN2* encode the carboxylic acid transporters (particularly mono- and dicarboxylic acids such as lactate, pyruvate, α-ketoglutarate [α-KG], and malate) that are strongly induced after phagocytosis by macrophages or neutrophils at the protein and RNA level, but the double deletion *jen1Δ jen2Δ* strain, which is incapable of importing several carboxylic acids, has been reported to show no defects in a disseminated candidiasis mouse model (18, 31, 32). Mutants lacking *STP2*, encoding a transcription factor that regulates amino acid uptake and utilization, are unable to neutralize the acidic environment and are modestly attenuated in both macrophage and mouse models (10). Mutants incapable of catabolizing GlcNAc are likewise modestly attenuated in macrophages and mice (13, 33).

The modest individual phenotypes led us to hypothesize that C. albicans simultaneously utilizes all of these nutrients during infection, with each carbon source providing unique benefits to the pathogen. Combining these effects through genetic elimination of multiple carbon pathways would thus have additive effects on fitness in macrophage interactions and disseminated infections. In support of this hypothesis, we report here that alternative carbon sources serve as unique priming signals with respect to the host environments that C. albicans may encounter. Each alternative carbon source induces a unique pattern of protection from host-relevant stressors compared to glucose-grown cells. Exposure to these nutrients also alters interactions with immune cells, likely as a result of significant changes in the fungal cell wall. Additionally, mutant strains lacking multiple carbon utilization pathways are significantly attenuated in macrophages and in mouse models. We conclude that C. albicans makes use of multiple alternative carbon sources simultaneously and that these serve as more than energy sources for the pathogen, each conferring unique benefits that allow it to adapt and cause infection in a variety of host environments.

## RESULTS

### Alternative carbon sources affect C. albicans stress tolerance.

Abundant evidence suggests that C. albicans utilizes multiple carbon sources *in vivo*, including glucose, carboxylic acids (lactate, α-ketoglutarate), amino acids, and GlcNAc, and that the catabolism of these compounds has effects beyond generation of energy and biomass (10, 13, 14, 18, 22, 26–28, 33). In particular, lactate induces changes to C. albicans cell wall morphology, stress resistance, and immune interactions that promote pathogenesis (15, 25–27). We asked whether these physiological adaptations might be triggered by additional alternative carbon sources, and whether the responses were carbon source specific.

**FIG 1:**
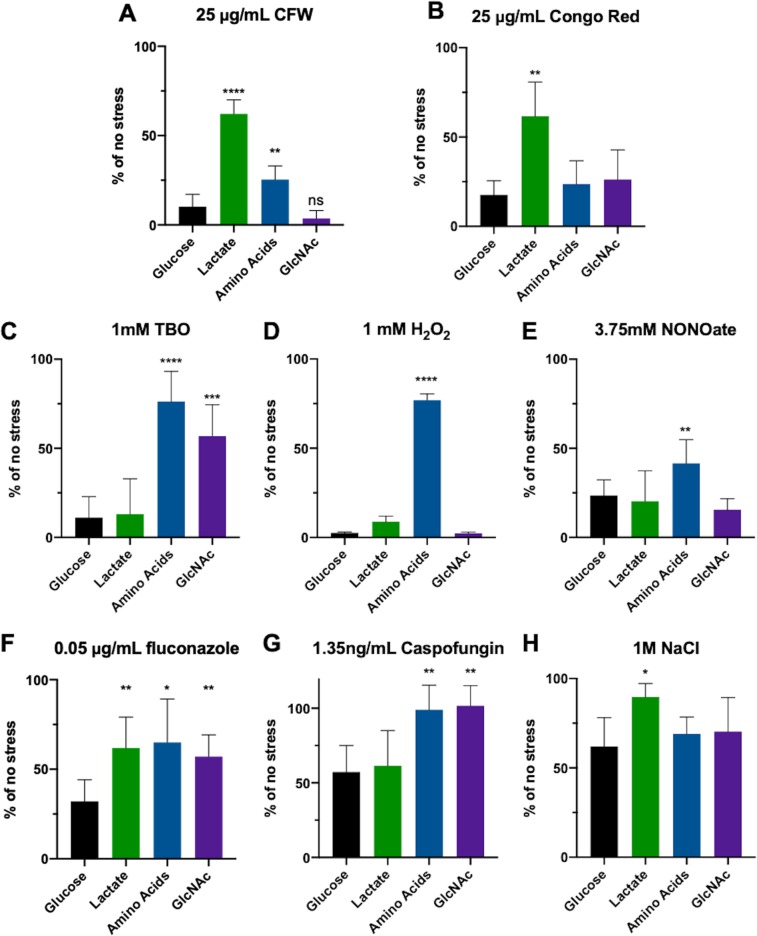
Alternative carbon utilization promotes resistance to a diverse array of stressors. SC5314 was grown for 6 h in minimal media containing 1% glucose or containing 1% glucose plus 1% of the indicated alternative carbon source and then transferred to the same media with or without the indicated stressor in a 96-well plate and incubated at 37°C with aeration in a plate reader for 16 h. The data represent the difference in the change in OD_600_ at the 8-h time point, expressed as a ratio of the level seen the presence of stress to that seen in the absence of stress. *N* ≥ 5 for each stress condition. *, *P* < 0.05; **, *P* < 0.01; ***, *P* < 0.001; ****, *P* < 0.0001 (unpaired *t* tests).

C. albicans encounters multiple stressors, including oxidative, nitrosative, osmotic, cell wall, and antifungal stresses, during disseminated infection. To assess how alternative carbon utilization affects host-relevant stress resistance, C. albicans cells were pregrown in minimal yeast nitrogen base with allantoin (YNBA) media containing 1% glucose or containing 1% glucose plus 1% of one of the three alternative sources, namely, lactate, Casamino Acids (CAA), or GlcNAc. Cells were then transferred to fresh media with or without the indicated stress. Growth was assessed in multiwell plates over time at 37°C in a multifunctional plate reader and quantified by assessing the change in optical density at 600 nm (OD_600_), relative to the no-stress control, after 8 h (Fig. 1; representative growth curves are presented in Fig. S1 in the supplemental material). In agreement with previous reports (15, 27), lactate protected C. albicans from high concentrations of the cell wall stressors calcofluor white (CFW) and Congo red (Fig. 1A and B). In contrast, lactate did not alter sensitivity to oxidative or nitrosative stresses (Fig. 1C to E). Lactate induced protection from the antifungal drug fluconazole but not from caspofungin (Fig. 1F and G). Similarly to previous reports, lactate also increased resistance to salt stress (Fig. 1H) (15).

10.1128/mBio.03070-19.1 FIG S1 Alternative carbon utilization promotes stress resistance. SC5314 was grown to mid-log phase in minimal media containing 1% glucose or containing 1% glucose plus 1% of the indicated carbon source. Cultures were washed and transferred to a 96-well plate in the same media with or without the indicated stressor. Dark curves indicate positive (+) stress conditions and light curves negative (−) stress conditions. *n* > 5; the displayed curves represent means. Download FIG S1, PDF file, 0.6 MB. Copyright © 2020 Williams and Lorenz. 2020 Williams and Lorenz This content is distributed under the terms of the Creative Commons Attribution 4.0 International license.

Interestingly, amino acids and GlcNAc also induced resistance to some of these stresses, but in distinct patterns: amino acids strongly induced protection from oxidative stressors *tert*-butyl-hydroperoxide (TBO) and H_2_O_2_ (Fig. 1C and D) and more modestly induced protection from nitrosative stress and CFW (Fig. 1A and H). It had no effect on Congo Red or nitrosative or osmotic stress (Fig. 1B, E, and H). In contrast, GlcNAc enhanced resistance to TBO but not peroxide (Fig. 1C and D) and had no effect on nitrosative, cell wall, or osmotic stress (Fig. 1A, B, E, and H). All three alternative carbon sources promoted fluconazole resistance, whereas amino acids and GlcNAc, but not lactate, protected cells from caspofungin (Fig. 1F and G).

As a corollary, we also asked whether alternative carbon sources provided protection against acute toxicity after shorter-term exposure to extreme stress concentrations. The proportion of cells grown under all three conditions that survived exposure to 10 mM TBO was much greater than that seen with glucose alone (Fig. S2). In contrast, only lactate protected against 2 M NaCl. Together, these data suggest that the changes in stress resistance represented an active response to the presence of these compounds and not a consequence of glucose deprivation. Moreover, each alternative carbon pathway was found to be physiologically distinct, inducing different patterns of stress resistance, which raises the possibility that nutritional composition is a signal that may allow C. albicans to anticipate likely stresses in specific host environments.

10.1128/mBio.03070-19.2 FIG S2 Fungal survival after challenge with high concentrations of stressors. (A) Green fluorescent protein (GFP)-expressing C. albicans cells were pregrown in minimal media containing 1% of the indicated carbon source for 6 h and then incubated with propidium iodide–10 mM TBO–PBS and imaged every hour using an imaging plate reader. Cell death was quantified by calculating the total number of red fluorescent cells (dead) divided by the total number of green fluorescent cells. *n* = 3. (B) Cells were pregrown as in described for A before incubation in 2 M NaCl or H_2_O for 1 h. Survival was determined by plating for CFU on YPD medium. *n* = 3. Download FIG S2, PDF file, 0.05 MB. Copyright © 2020 Williams and Lorenz. 2020 Williams and Lorenz This content is distributed under the terms of the Creative Commons Attribution 4.0 International license.

### Alternative carbon sources alter interactions with macrophages.

**FIG 2:**
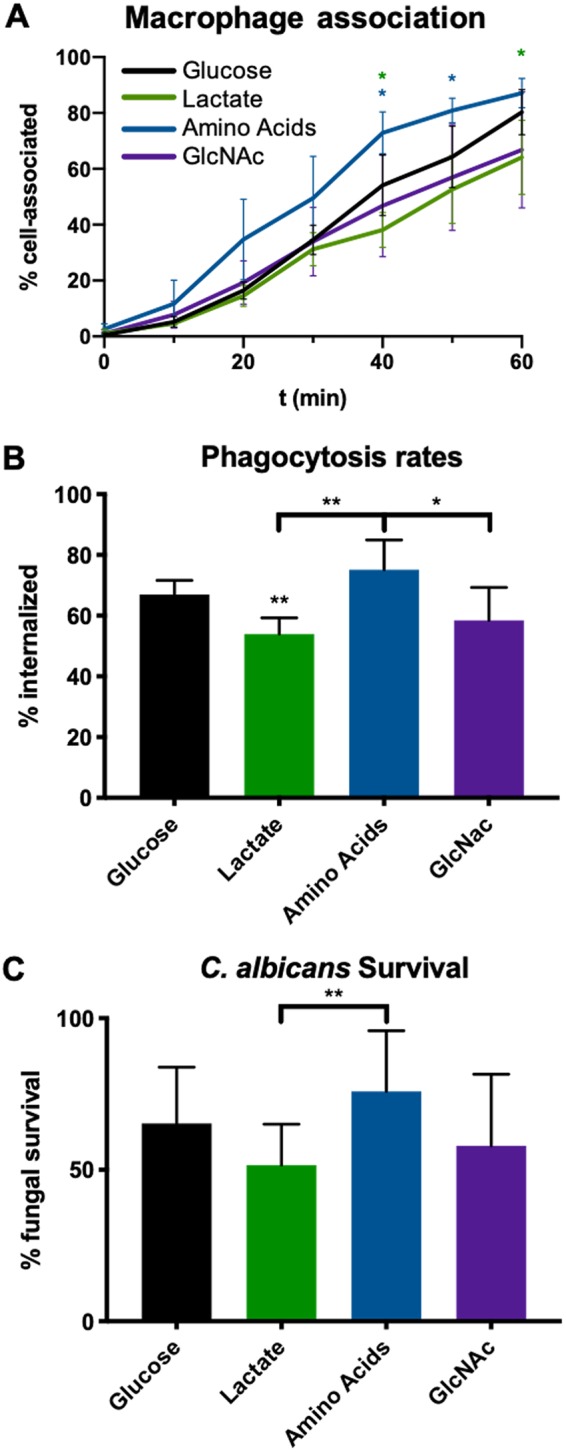
Alternative nutrients affect C. albicans interactions with macrophages. SC5314 was grown for 6 h in minimal media containing 1% of the indicated carbon source prior to each experiment. (A) SC5314 expressing scarlet fluorescent protein was coincubated with J774A macrophages stained with nucBlue and actin green in DMEMat 5% CO_2_ and 37°C and was imaged every 15 min in an imaging plate reader. Cell association was quantified as the number of red C. albicans cells that were overlaid with green macrophages. *n* = 5. (B) Similarly to the method described for panel A, scarlet fluorescent protein-expressing SC5314 was coincubated with J774 macrophages stained with DAPI (4′,6-diamidino-2-phenylindole) for 60 min. Samples were fixed with paraformaldehyde and counterstained with FITC to identify external fungal cells. *n* = 5. (C) Fungal survival was assessed after 16 h of coincubation with J774 macrophages using a modified CFU assay. Percent survival data are compared to data from a control well containing C. albicans strains incubated without macrophages. *n* = 5. *, *P* < 0.05; **, *P* < 0.01; ***, *P* < 0.001; ****, *P* < 0.0001 (unpaired *t* tests).

Lactate exposure alters interactions with macrophages, but little is known about the effects of amino acids and GlcNAc (27). C. albicans was again pregrown to mid-log phase in minimal media containing glucose with or without the alternative carbon sources before assessing macrophage interactions. Media were buffered to pH 5.5 to prevent the cell wall changes that can occur under conditions of pH fluctuations (30). As previously reported, the presence of lactate modestly decreased the rate at which cells associated with and were phagocytosed by macrophages (Fig. 2A and B). Interestingly, pregrowth in amino acids resulted in increased phagocytosis rates compared to those seen with glucose-grown cells, while GlcNAc had no significant effect. Despite the increased phagocytosis, the amino acid-grown cells showed significantly improved fungal survival compared to the lactate-exposed cells, but the differences from the glucose-grown cells were not statistically significant (Fig. 2C). Together, these data show that alternative carbon sources, particularly lactate and amino acids, distinctly affect immune recognition of fungal cells.

C. albicans occupies a phagolysosome of significantly higher pH than would be expected, and we have previously found that this is associated with utilization of alternative carbon sources (10, 14, 34, 35). We next asked whether prior exposure to these nutrients would affect the ability to neutralize the pH of macrophage phagosomes. To address this issue, we loaded macrophages with the acidophilic dye LysoTracker red prior to culturing with C. albicans cells (pregrown as described above) in media containing glucose, lactate, amino acids, or GlcNAc. The level of phagosome acidity immediately surrounding the fungal cell was calculated by measuring LysoTracker intensity immediately outside the fungal cell, as previously described (11, 13). Interestingly, no differences were observed in the levels of phagosome acidity after exposure to alternative carbon sources (Fig. S3).

10.1128/mBio.03070-19.3 FIG S3 Pregrowth in alternative carbon sources does not affect the ability of C. albicans to neutralize the phagosome. Phagosomal pH was estimated using LysoTracker Red staining as previously described (Vylkova and Lorenz [14]). In short, macrophages preloaded with acidophilic dye were coincubated with C. albicans for 1 h and fixed, and fluorescent microscopy was performed. Prior to coincubation, C. albicans was grown for 6 h in YNBA medium plus 1% of the indicated carbon source buffered to pH 5.5. Red fluorescence intensity is reflective of an acidic phagosome. *n* = 3. Download FIG S3, PDF file, 0.10 MB. Copyright © 2020 Williams and Lorenz. 2020 Williams and Lorenz This content is distributed under the terms of the Creative Commons Attribution 4.0 International license.

### Alternative carbon sources alter cell wall structure.

**FIG 3:**
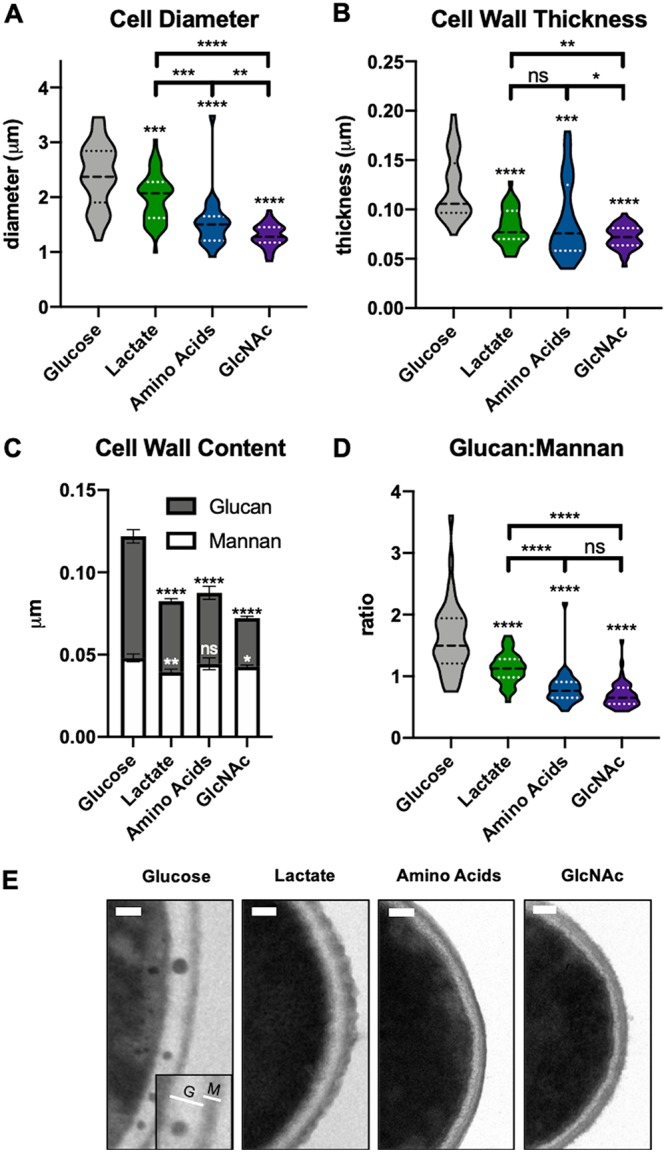
Carbon source affects cell wall morphology. Wild-type cells were grown at 37°C to mid-log phase in minimal media containing 1% of the indicated carbon source and buffered to pH 5.5. Cultures were fixed in Karnovsky’s fixative and then processed to perform transmission electron microscopy. (A) Yeast diameters were measured twice per cell and averaged. *n* ≥ 50 per condition. (B) Cell wall thickness. At least 15 measurements were performed per cell. *n* ≥ 50 per condition. (C) Thickness of glucan and mannan layers. (D) Glucan/mannan ratio under each condition. (E) Representative images representing each condition. The inset in the “Glucose” panel represents an example of the regions measured for the glucan (G) and mannan (M) layers of the wall. Scale bar = 0.1 μm. *, *P* < 0.05; **, *P* < 0.01; ***, *P* < 0.001; ****, *P* < 0.0001 (unpaired *t* tests).

The changes observed in response to the presence of lactate were found to be associated with alterations to cell wall structure (15). To assess whether amino acids and GlcNAc had similar effects on the cell wall, we visualized this organelle using transmission electron microscopy (Fig. 3). C. albicans was grown to mid-log phase in minimal medium (YNBA) with a carbon source (glucose, Casamino Acids, lactate, or GlcNAc) (1%) buffered to pH 5.5 before fixing and processing. The diameter of yeast-phase cells grown on alternative carbon sources was significantly reduced compared to that of glucose-grown cells (Fig. 3A). This is in line with observations in both single-celled and multicellular organisms indicating that nutrient restriction generally results in reduced cell size (36–39). Growth in alternative carbon sources reduced the overall thickness of the cell wall (Fig. 3B); the inner glucan layer was markedly thinner, while the outer mannan layer was mostly unchanged (Fig. 3C). As a result, the ratio of the thicknesses of the glucan and mannan layers was greatly reduced in cells grown in the absence of glucose (Fig. 3D). Notably, the glucan layer of both amino acid-grown and GlcNAc-grown cells was more electron dense than that of glucose-grown and lactate-grown cells (Fig. 3E). Electron-dense granules of 30 to 100 nm were frequently seen in the cell walls of glucose-grown cells but not under the other conditions (Fig. 3E); we assume that they represent extracellular vesicles transiting the wall. Thus, utilization of lactate, GlcNAc, and amino acids results in distinct decreases in cell size and cell wall thickness, shifts in the ratios of the thicknesses of the glucan and mannan layers, and alterations in cell wall density.

### Strains unable to utilize alternative carbon sources are defective in multiple fitness assays.

**FIG 4:**
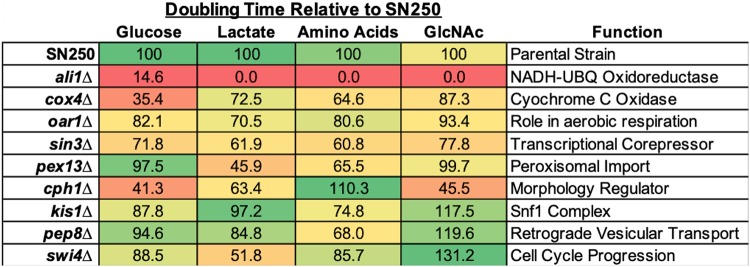
Homann and Noble library mutants identified via genetic screens display growth defects on multiple carbon sources. Mutants identified via genetic screens were grown in minimal media plus 1% alternative carbon source. Mid-log-phase doubling times were calculated and expressed as a percentage of the rate determined for the parental strain (SN250) in the same media. Values displayed represent averages of results from 3 replicates.

Since exposure of C. albicans to individual alternative carbon sources has overlapping effects on cell wall morphology, stress resistance, and interactions with macrophages, we next asked whether genetically interfering with alternative carbon utilization would decrease fitness within the host. We conducted a genetic screen for mutants that grow poorly on media containing lactate as the sole carbon source, using available libraries of ∼850 strains (the Noble and Homann libraries) (40, 41). The libraries were grown in yeast extract-peptone-dextrose (YPD) medium and transferred to minimal medium containing 1% lactate (plus 10 mM arginine, as the library strains are arginine auxotrophs) and assessed for growth and pH neutralization using the pH indicator bromocresol purple. Hits from this screen were combined with those from a previously reported screen for mutants impaired on the same media with α-ketoglutarate (10). Mutants with initial growth defects were confirmed via PCR and converted to prototrophy using CIp30, and doubling times were assessed in multiple media (Fig. 4).

These mutants had a spectrum of phenotypes (Fig. 4), with some strains impaired in all carbon sources and others in a subset. A few strains (*ali1Δ*, *cox4Δ*, *sin3Δ* mutants) also conferred significant growth defects in glucose-containing minimal media, as has been reported previously (40, 42). Unsurprisingly, many of the mutants encoded proteins associated with functions required for aerobic respiration. These include Cox4 (a subunit of cytochrome *c*), Ali1 (a membrane-bound NADH-ubiquinone oxidoreductase), and Oar1 (a putative mitochondrial protein whose homologs have NADPH-dependent 3-oxoacyl reductase activity) (40, 42). Two mutants have known roles in other aspects of carbon metabolism, including the Snf1 complex protein Kis1 and Pex13, which is required for protein import to the peroxisome (43–45). Others had no clear connection to carbon utilization, including Pep8 (a protein involved in endosome-to-Golgi transport), Swi4 (part of the SBF complex required for the G_1_/S transition), and Sin3 (a global transcriptional corepressor) (46–49). The well-characterized morphology regulator Cph1 also has defects in growth on alternative carbon sources, though this particular library mutant also grew more slowly when glucose was present, which is not a phenotype that was previously associated with the *cph1Δ* mutation. Notably, the *cox4Δ* mutant was able to grow in GlcNAc but did so without forming hyphae.

**FIG 5:**
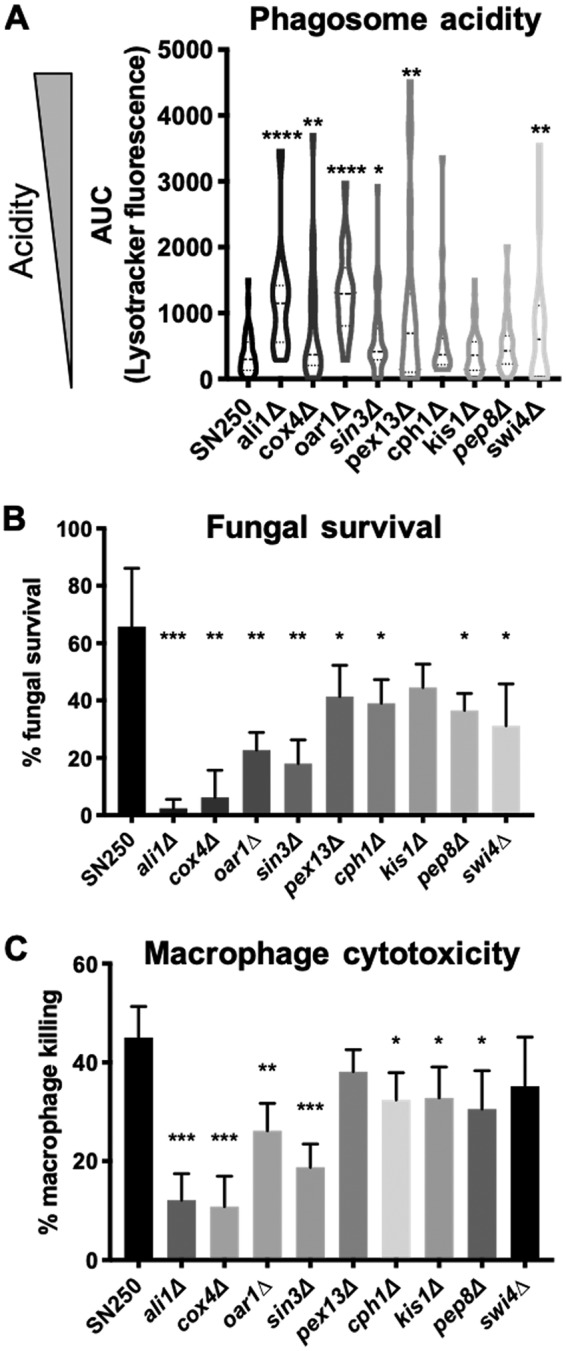
Multiple genetic screen mutants are defective within macrophages. (A) Phagosomal pH was estimated using LysoTracker Red staining as previously described (Vylkova and Lorenz [14]). In short, macrophages preloaded with acidophilic dye were coincubated with C. albicans for 1 h and fixed, and fluorescence microscopy was performed. Red fluorescence intensity is reflective of an acidic phagosome. *n*  = 3. (B) Fungal survival quantified via modified CFU assay as described for Fig. 3. *n* = 4. (C) Macrophage survival was quantified via lactate dehydrogenase release after 16 h of coincubation with the indicated C. albicans strain. Killing data are compared to results determined for chemically lysed macrophages. *n* = 4. *, *P* < 0.05; **, *P* < 0.01; ***, *P* < 0.001; ****, *P* < 0.0001 (unpaired *t* tests).

These mutants were evaluated for fitness defects in multiple macrophage assays (Fig. 5). Since alternative carbon sources promote extracellular pH neutralization, we assessed the ability of the mutants to neutralize the phagosome. Several mutants occupied significantly more acidic phagosomes within J774A.1 cells, indicative of pH neutralization defects (Fig. 5A). Failure to neutralize the phagosome was previously shown to be associated with impaired fungal survival in macrophages, and, indeed, all of the screened mutants, with the possible exception of the *kis1Δ* strain (where the decrease was not statistically significant), survived a 16-h coincubation less readily than the control strain (Fig. 5B). Among these mutants, the *ali1Δ*, *cox4Δ*, and *oar1Δ* strains also showed notable hyphal defects within the macrophage. Th*e cox4Δ* mutant appeared to be entirely incapable of forming hyphae, as it was found in the yeast and pseudohyphal forms under conditions that strongly induced hypha (RPMI plus serum, 5% CO_2_, 37°C) (data not shown). We also tested macrophage survival, using release of cytosolic lactate dehydrogenase (LDH) as a proxy for membrane damage, and found that all of the mutants, with the possible exception of the *pex13Δ* and *swi4Δ* strains, caused less damage to macrophages (Fig. 5C). Thus, among multiple mutants with defects in utilization of alternative carbon sources, nearly all were attenuated in multiple aspects of interactions with macrophage, further indicating the importance of alternative carbon utilization during infection.

### Engineered carbon-deficient mutants are impaired in multiple virulence models.

**FIG 6:**
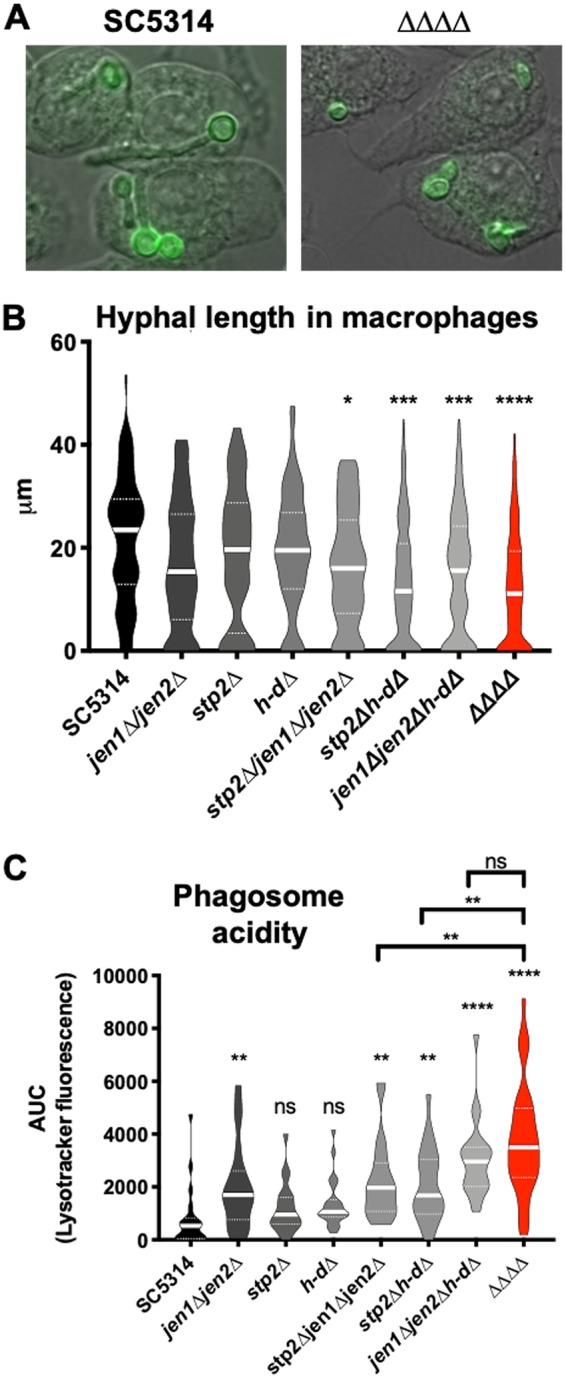
Alternative carbon mutants are defective upon macrophage phagocytosis. (A and B) FITC-labeled strains were cocultured with J774 macrophages in DMEM for 2 h. Cells were fixed and stained with calcofluor white, and germ tube lengths were quantified in triplicate experiments (>40 per strain per replicate). Scale bar = 10 μm. (C) A LysoTracker Red assay was performed in a manner similar to that described for Fig. 5. *n* = 3. *, *P* < 0.05; **, *P* < 0.01; ***, *P* < 0.001; ****, *P* < 0.0001 (unpaired *t* tests).

As a second and more direct approach to address this issue, we employed the CRISPR-Cas9 system to generate strains lacking one or more of the genes that have been previously shown to directly contribute to nutrient import and catabolism, i.e., *STP2*, *JEN1*, *JEN2*, *HXK1*, *NAG1*, and *DAC1* (11, 29, 32). Stp2 is a transcription factor that controls amino acid import and catabolism. Jen1 and Jen2 are mono- and dicarboxylic acid transporters, respectively. Hxk1, Nag1, and Dac1 are three enzymes essential for GlcNAc catabolism and are encoded by a cluster of adjacent genes on chromosome 6 such that they could be knocked out in tandem (“*h-dΔ*”) (50). Individually, these mutants were previously shown to have modest impacts on fungal survival in macrophages and virulence in a disseminated mouse model (13, 14, 32). We hypothesized that these phenotypes would be additive, such that elimination of multiple carbon pathways would result in markedly increased attenuation. We thus constructed the *stp2Δ*, *jen1Δ jen2Δ*, and *h-dΔ* mutant strains and all possible combinations thereof, including the quadruple mutant, termed the “ΔΔΔΔ” strain. This strain was unable to grow on YNBA media containing lactate (monocarboxylic acid), α-ketoglutarate (dicarboxylic acid), Casamino Acids, or GlcNAc and did not neutralize the pH of these media (Fig. S4). The quadruple mutant grew at wild-type rates in YNBA-glucose or YPD media, germinated normally under conditions that strongly induced hypha (Fig. 6A; see also Fig. S5), and displayed no apparent differences in macrophage uptake rates. Additionally, supplementing the media with glycerol partially rescued growth but not pH changes, suggesting that the neutralization is dependent on alternative carbon catabolism processes (Fig. S6). Thus, we successfully generated a strain that was incapable of using multiple alternative carbon sources but that appeared not to have pleiotropic phenotypes.

10.1128/mBio.03070-19.4 FIG S4 The ΔΔΔΔ strain did not grow or neutralize the pH of minimal media containing alternative carbon sources. Growth (left) and medium pH (right) of wild-type C. albicans (SC5314, black) and the ΔΔΔΔ strain (red) in YNBA medium plus glucose or an alternative carbon source. (A) Glucose. (B) Lactate. (C) α-Ketoglutarate (α-KG). (D) Casamino Acids. (E) GlcNAc. Growth curve analyses were performed in triplicate. The inset in the graph at the left in panel E shows GlcNAc-grown cells after 6 h. Cell clumping prevented accurate measurement of OD_600_. Download FIG S4, PDF file, 0.4 MB. Copyright © 2020 Williams and Lorenz. 2020 Williams and Lorenz This content is distributed under the terms of the Creative Commons Attribution 4.0 International license.

10.1128/mBio.03070-19.5 FIG S5 The ΔΔΔΔ strain has no hyphal defects. Strains were grown to mid-log phase in YPD medium then incubated under conditions of strong hypha induction (RPMI medium plus 5% serum, 37°C, 5% CO_2_). Hyphal formation was visualized every 30 min for 12 h. Images shown are from the 6-h time point. Download FIG S5, PDF file, 2.3 MB. Copyright © 2020 Williams and Lorenz. 2020 Williams and Lorenz This content is distributed under the terms of the Creative Commons Attribution 4.0 International license.

10.1128/mBio.03070-19.6 FIG S6 Supplementation of growth with glycerol does not rescue pH neutralization phenotype with the ΔΔΔΔ strain. The SC5314 (black) and ΔΔΔΔ (red) strains were grown in YNBA medium plus 1% alternative carbon source and 1% glycerol (unbuffered) to pH 4.0. *n* = 3. Download FIG S6, PDF file, 0.04 MB. Copyright © 2020 Williams and Lorenz. 2020 Williams and Lorenz This content is distributed under the terms of the Creative Commons Attribution 4.0 International license.

Using strains defective in one, two, and three carbon pathways (including strain ΔΔΔΔ and all intermediate strains), we assayed fitness after phagocytosis by macrophages to determine if the multiple mutants had additive defects (Fig. 6). After 2 h of coculture, each single mutant trended toward reduced hyphal lengths, while the multiple mutants showed significantly reduced hyphal elongation (Fig. 6A and B). Each carbon pathway has been associated with a reduced ability to neutralize the phagolysosome, and strains impaired in two or all three pathways are found in even more acidic phagosomes (Fig. 6C). Hyphal length and phagosomal pH are correlated, though it is not clear whether neutral pH induces germination or whether hyphal growth neutralizes the phagosome through membrane disruption (14, 51). These data suggest that C. albicans utilizes multiple alternative carbon sources simultaneously within a macrophage phagosome, such that these pathways are partially redundant in supporting both energy generation and the substantial biomass addition necessary for hyphal growth and subsequent escape.

**FIG 7:**
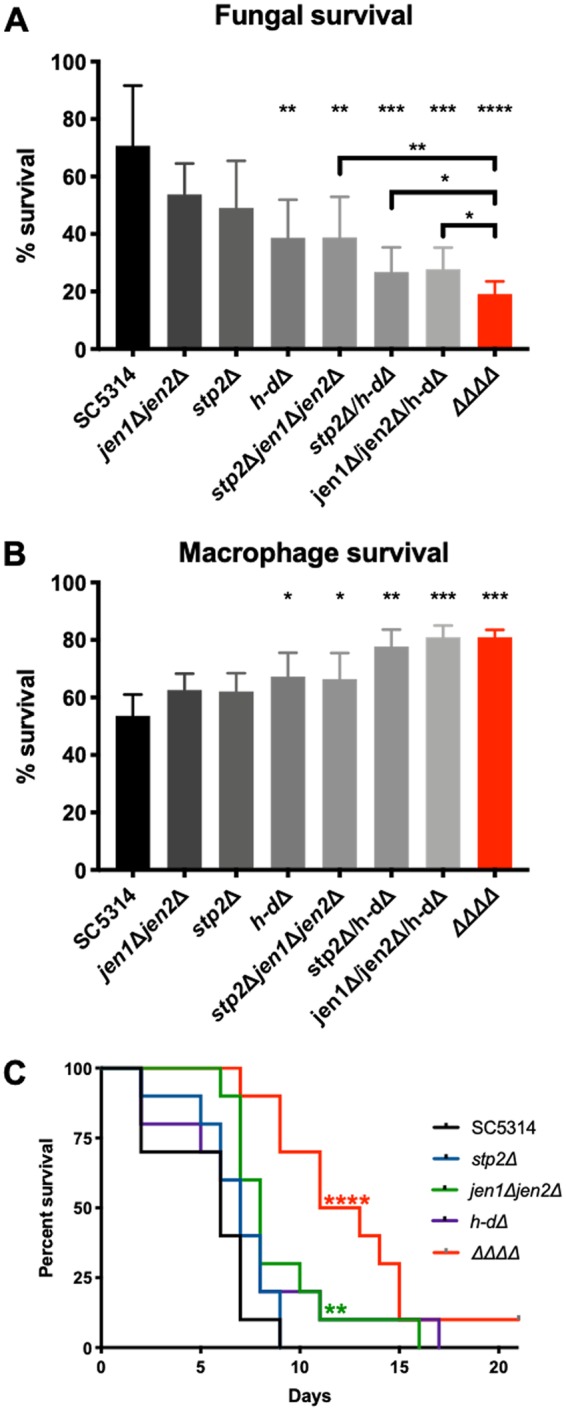
Alternative carbon mutants are attenuated in both macrophage and mouse models for disseminated candidiasis. (A) Fungal survival was assessed after 16 h of coincubation with J774 macrophages using a modified CFU assay. *n* = 7. (B) Macrophage survival was quantified via analysis of LDH release after 16 h of coincubation with the indicated C. albicans strain. Survival rates are relative to the results seen with chemically lysed macrophages. MOI 1:1; *n* = 4. (C) Outbred ICR mice were injected via tail vein injection with 5 × 10^5^ cells/ml of the indicated strains resuspended in phosphate-buffered saline (10 mice per strain). *, *P* < 0.05; **, *P* < 0.01; ***, *P* < 0.001; ****, *P* < 0.0001 (unpaired *t* tests), for panels A and B. Survival curves were compared using the log rank (Mantel-Cox) test.

We next evaluated whether the alternative carbon mutants would show compromised fitness in the macrophage model, assessing both fungal and macrophage survival after a 16-h coincubation (Fig. 7A and B). In alignment with the hyphal and phagosome acidity phenotypes, the levels of fitness of the alternative carbon mutants were found to be increasingly attenuated as more carbon pathways were eliminated, with the quadruple mutant the most severely impaired. This again suggests that C. albicans utilizes many different nutrient sources upon phagocytosis, a departure from the general paradigm of carbon metabolism in fungi.

Finally, we assessed the virulence of the single and quadruple mutant strains in the mouse intravenous model of disseminated candidiasis. In this model, the virulence of the quadruple mutant was the most severely attenuated compared to the parental strain, and this difference was significantly different from the results seen with the *stp2Δ* and *h-dΔ* strains. In this model, the virulence of the *jen1Δ jen2Δ* strain was slightly attenuated relative to the wild-type strain (the statistical significance was probably the result of the presence of a single outlier that survived much longer than the rest of the members of that experimental group). This contrasts with the macrophage model, in which the *jen1Δ jen2Δ* strain was the least affected, again pointing to niche-specific differences in carbon availability and importance. Altogether, these data show that C. albicans actively utilizes multiple carbon sources during disseminated infection, which changes fungal morphology, alters stress resistance and immune interactions, and ultimately is essential for pathogenesis.

## DISCUSSION

Several lines of evidence demonstrate that the metabolism of nonglucose carbon sources is critical during interactions of C. albicans with phagocytes and in animal models. Many transcriptomic data sets confirmed the occurrence of a wholesale metabolic change upon phagocytosis, with the cell switching from glycolysis to gluconeogenesis (18, 20, 52). This response is not specific to *Candida*, as many other fungal pathogens, including Cryptococcus neoformans, Aspergillus fumigatus, Talaromyces marneffei, and Paracoccidioides brasiliensis also adapt their metabolism to gain advantages over the host (53–56). Gluconeogenic genes are also induced *in vivo* (21, 57), and these transcriptional changes are often advantageous for microbial pathogens. Stress resistance induced in the presence of alternative carbon sources has been observed in other *Candida* species, including C. glabrata and the emerging pathogen C. auris (58, 59), and numerous examples of beneficial effects of utilization of alternative carbon sources have been seen in bacterial pathogens. For example, Staphylococcus aureus, a facultative intracellular pathogen, alters its central carbon metabolism to promote its survival in the phagosome (60, 61). The ability to utilize short-chain fatty acids is required for full virulence of Campylobacter jejuni (62), and Mycobacterium tuberculosis requires the glyoxylate shunt for survival in macrophages and mice (63, 64). Here, we show that, similarly to many other pathogens, C. albicans appropriately responds to multiple alternative carbon sources to gain advantages over the host.

Mutants impaired in central carbon catabolism via the tricarboxylic acid (TCA)/glyoxylate cycle (*icl1Δ* strain) or via gluconeogenesis (*pck1Δ* and *fbp1Δ* strains) have also been found to be attenuated in macrophage and mouse models (21, 23). Understanding which specific nutrients are most relevant *in vivo* may identify candidate drug targets, as some secondary carbon metabolic pathways, such as the glyoxylate cycle pathway and some amino acid biosynthetic pathways, do not exist or are substantially divergent in mammals. To this end, mutants were previously generated in specific uptake or catabolic pathways, such as those for fatty acids (strain *fox2Δ*), amino acids (strain *stp2Δ*), GlcNAc (strain *h-dΔ*), oligopeptides (strain *optΔ ptrΔ*), or carboxylic acids (strain *jen1Δ jen2Δ*), and these have modest or no *in vivo* phenotypes (19, 23, 32, 33, 65, 66). In contrast, the attenuation of strain ΔΔΔΔ (*stp2Δ jen1Δ jen2Δ h-dΔ*) in macrophage and mouse models affirms that alternative carbon utilization directly contributes to fungal pathogenesis, and the additive effects observed suggest that C. albicans utilizes multiple carbon sources simultaneously within the macrophage phagosome and during disseminated infection, though we cannot exclude the possibility that the observed differences in virulence resulted in part from other changes, such as changes to the cell wall, induced by variations in nutrient availability.

In addition to the energy generation that is provided by utilizing alternative carbon sources, carboxylic acids, amino acids, and GlcNAc serve as unique signals for C. albicans, modulating stress resistance and macrophage interactions. This was first recognized with lactate, where drug and stress resistance correlated with alterations in the cell wall (25, 27, 28). Amino acids and GlcNAc also induce cell wall alterations and promote stress resistance. Surprisingly, each carbon source confers a unique pattern of resistance; amino acids confer resistance to oxidative and nitrosative stresses, lactate confers resistance to cell wall and osmotic stresses, and GlcNAc confers resistance to oxidative stress. Transcriptomic data provide some basis for these patterns; amino acid-grown cells upregulate several oxidative and nitrosative stress resistance genes, including *SOD4* and *YHB1* (10), and *SOD5* is upregulated by GlcNAc (33). Antifungal resistance to fluconazole is also enhanced by all three classes of compounds and to caspofungin by GlcNAc and amino acids. The abundance of these nutrients in the host differs from site to site, as does the spectrum of immune cells and microbial competition (22). This raises the possibility that C. albicans uses nutrient availability as a signal of its anatomical niche, priming the cell for different arrays of encounters depending on location.

Indeed, C. albicans is equipped with sensors for all three alternative carbon pathways. Lactate sensing with Gpr1 is required for β-glucan masking and immune evasion, while import and catabolism are dispensable (26). The plasma membrane SPS amino acid sensor (Ssy1, Ptr3, Ssy5) regulates Stp2 and has very similar macrophage phenotypes (67), but it is not clear whether sensing and metabolism can be decoupled. GlcNac sensing (by Ngs1) and import (through Ngt1) are required for hyphal induction (29), suggesting that catabolism may also be dispensable for GlcNAc-induced stress resistance. Separating nutrient sensing and catabolism for pathogenesis and stress resistance will provide new insights into how pathogenic fungi detect, interact with, and adapt to various host environments.

In addition to the carbon sources studied here, there are likely several other carbon sources that promote fungal survival within the host. β-Oxidation is highly upregulated within macrophages, suggesting that fatty acids such as oleic acid are utilized within the host, although disruption of β-oxidation (*fox2Δ*) has a minor impact on pathogenesis (23, 24, 68). We mainly focused on the carboxylic acid lactate in this study on the basis of precedents, but preliminary data suggest that C. albicans responds differentially to additional host-relevant carboxylic acids such as α-ketoglutarate and acetate (15, 25, 27). Host-associated sugars besides GlcNAc (sialic acid, galactosamine), in addition to other lipids (e.g., sphingolipids), may be recognized as well.

In conclusion, this report highlights the importance of nutrient flexibility for fungal pathogenesis. In addition to serving as an energy source, alternative carbon sources prime C. albicans to tolerate challenging host environments, ultimately allowing the fungal pathogen to inhabit, survive in, and effectively infect a variety of host niches. Together, these host-relevant carbon sources serve as both an energy source and a signal, enabling C. albicans to undergo the virulence adaptations required for pathogenesis.

## MATERIALS AND METHODS

### Strains and media.

All strains used in this study are listed in Table 1. For general growth and propagation, C. albicans strains were grown in yeast extract-peptone-dextrose (YPD) medium (1% yeast extract, 2% peptone, 2% glucose, 2% agar for solid medium) at 30°C. YPD plus 200μg/ml nourseothricin (Werner Bioagents, Jena, Germany) was used to select for deletion mutants, and YP maltose (YPM) induced flipping out of the nourseothricin cassette as described previously (69).

**TABLE 1:** *Candida albicans* strains^*a*^

Strain	Description	Complete genotype	Reference or source
SC5314	Wild type	Prototroph	75
SN250	Library control + *Cip30*	*his1Δ*::*hisG*/*his1Δ*::*hisD leu2Δ*::*CdHIS1*/*leu2Δ*::*CMLEU2 arg4Δ*::*hisG*/*arg4Δ*::*hisG RPS10*/*rps10*:: *CIp30-ARG4*	This study
MG01	Constitutive scarlet		This study
RBW16	*ali1Δ + CIp30*	*ali1Δ*::*HIS1*/*ali1Δ*::*LEU2 his1*/*his1 leu2*/*leu2 arg4*/*arg4 RPS10*/*rps10*::*CIp30-URA3-HIS1-ARG4*	This study
RBW17	*cox4Δ + CIp30*	*cox4Δ*::*HIS1*/*cox4Δ*::*LEU2 his1*/*his1 leu2*/*leu2 arg4*/*arg4 RPS10*/*rps10*::*CIp30-URA3-HIS1-ARG4*	This study
RBW18	*oar1Δ + CIp30*	*oar1Δ*::*HIS1*/*oar1Δ*::*LEU2 his1*/*his1 leu2*/*leu2 arg4*/*arg4 RPS10*/*rps10*::*CIp30-URA3-HIS1-ARG4*	This study
RBW19	*sin3Δ + CIp30*	*sin3Δ*::*HIS1*/*sin3Δ*::*LEU2 his1*/*his1 leu2*/*leu2 arg4*/*arg4 RPS10*/*rps10*::*CIp30-URA3-HIS1-ARG4*	This study
C_emv14	*pex13Δ+ CIp30*	*pex13Δ*::*HIS1*/*pex13Δ*::*LEU2 his1*/*his1 leu2*/*leu2 arg4*/*arg4 RPS10*/*rps10*::*CIp30-URA3-HIS1-ARG4*	This study
RBW20	*cph1Δ + CIp30*	*cph1Δ*::*HIS1*/*cph1Δ*::*LEU2 his1*/*his1 leu2*/*leu2 arg4*/*arg4 RPS10*/*rps10*::*CIp30-URA3-HIS1-ARG4*	This study
RBW21	*kis1Δ + CIp30*	*kis1Δ*::*HIS1*/*kis1Δ*::*LEU2 his1*/*his1 leu2*/*leu2 arg4*/*arg4 RPS10*/*rps10*::*CIp30-URA3-HIS1-ARG4*	This study
RBW22	*pep8Δ + CIp30*	*pep8Δ*::*HIS1*/*pep8Δ*::*LEU2 his1*/*his1 leu2*/*leu2 arg4*/*arg4 RPS10*/*rps10*::*CIp30-URA3-HIS1-ARG4*	This study
RBW23	*swi4Δ + CIp30*	*swi4Δ*::*HIS1*/*swi4Δ*::*LEU2 his1*/*his1 leu2*/*leu2 arg4*/*arg4 RPS10*/*rps10*::*CIp30-URA3-HIS1-ARG4*	This study
RBW05	*jen1Δ jen2Δ*	*jen1Δ*::*FRT*/*jen1Δ*::*FRT jen2Δ*::*FRT*/*jen2Δ*::*FRT*	This study
RBW10	*stp2Δ*	*stp2Δ*::*FRT*/*stp2Δ*::*FRT*/*stp2Δ*::*FRT*	This study
RBW11	*hxk1*Δ *nag1* Δdac1Δ	*hxk1Δ nag1Δ dac1Δ*::*FRT*/*hxk1Δ nag1Δ dac1*::*FRT*	This study
RBW12	*stp2Δ jen1Δ jen2Δ*	*stp2Δ*::*FRT*/*stp2Δ*::*FRT*/*stp2Δ*::*FRT jen1Δ*::*FRT*/*jen1Δ*::*FRT jen2Δ*::*FRT*/*jen2Δ*::*FRT*	This study
RBW13	*stp2Δ hxk1Δ nag1Δ dac1Δ*	*stp2Δ*::*FRT*/*stp2Δ*::*FRT*/*stp2Δ*::*FRT hxk1Δ nag1Δ dac1Δ*::*FRT*/*hxk1Δ nag1Δ dac1*::*FRT*	This study
RBW14	*jen1Δ jen2Δ hxk1Δ nag1Δ* *dac1Δ*	*jen1Δ*::*FRT*/*jen1Δ*::*FRT jen2Δ*::*FRT*/*jen2Δ*::*FRT hxk1Δ nag1Δ dac1Δ*::*FRT*/*hxk1Δ nag1Δ dac1*::*FRT*	This study
RBW15	*jen1Δ jen2Δ stp2Δ hxk1Δ* *nag1Δ dac1Δ*	*jen1Δ*::*FRT*/*jen1Δ*::*FRT jen2Δ*::*FRT*/*jen2Δ*::*FRT stp2Δ*::*FRT*/*stp2Δ*::*FRT*/*stp2Δ*::*FRT hxk1Δ nag1Δ* *dac1Δ*::*FRT*/*hxk1Δ nag1Δ dac1*::*FRT*	This study

a Strains RBW16-23 and C_emv14 are prototrophic derivatives from the mutant library developed by Noble et al. (40).

Alternative carbon growth and pH experiments required the use of YNB medium with allantoin as the nitrogen source (0.17% yeast nitrogen base without ammonium sulfate and amino acids, 0.5% allantoin) and 1% of the indicated carbon source, adjusted to the indicated pH using HCl or NaOH, similarly to previous publications (10, 13). For the electron microscopy and stress resistance experiments, the medium was buffered to pH 5.5 with 25 mM HEPES.

J774A.1 murine macrophages were maintained in Dulbecco’s modified Eagle’s medium (DMEM) plus glutamate and 10% fetal bovine serum (FBS), supplemented with penicillin and streptomycin, at 37°C in a 5% CO_2_ humidified environment. DMEM without a pH indicator, penicillin/streptomycin (pen/strep), or FBS was used for the coculture assays.

### Strain construction.

All genetic deletions were generated using the CRISPR/Cas9 SAT-Flipper method (70, 71). Mutants were verified via PCR, and the maltose-inducible SAT cassette was flipped out in order to generate subsequent deletions in the same strain.

Auxotrophic screen mutants from the Noble and Homann libraries were transformed via electroporation with the CIp30 plasmid digested with StuI (40). All mutants were PCR verified to confirm appropriate gene deletion.

### Growth and pH neutralization assays.

Growth and pH changes were assayed as described previously (10, 14). Briefly, strains were grown overnight in YPD medium at 30°C, centrifuged, washed with YNBA media, and diluted into the YNBA media for a starting OD_600_ of 0.2. Samples were incubated with aeration for 24 h at 37°C, with growth (OD_600_) and pH assayed every 2 h. Assays were performed in at least triplicate.

### Transmission electron microscopy.

C. albicans was grown to mid-log phase in YNBA medium plus the indicated carbon source in 50 ml-flasks with shaking at 37°C and then pelleted by low-speed centrifugation, fixed overnight in Karnovsky’s fixative, and stored at 4°C until processing for electron microscopy was performed (72). Cells were postfixed in osmium tetroxide, dehydrated in a graded series of ethanol, and embedded in epoxy resin as described previously (73). Sections (100-nm thick) were stained with uranyl acetate and lead citrate. Electron micrographs were collected from randomly selected yeast cells with a JEOL JEM-1230 transmission electron microscope equipped with a digital charge-coupled-device (CCD) camera. All measurements were quantified with ImageJ. The thickness of the cell wall was determined by averaging at least 15 measurements around the wall of each cell, and at least 50 cells were analyzed per condition. Unpaired Student’s *t* tests were performed to determine statistical differences for all measurements.

### Stress resistance assays.

C. albicans was pregrown for 6 h in the indicated medium with rolling at 30°C and then transferred to a 96-well plate at a starting OD_600_ of 0.1. Growth in the presence or absence of the indicated stressor was assayed via OD_600_ determination every 15 min using a Cytation 5 plate reader (BioTek) with orbital shaking at 37°C. Growth differences were quantitated as ratios of the changes in OD_600_ after 8 h under the stress conditions to that in the n- stress control (percentage of no stress = ΔOD_600_[stress]/ΔOD_600_[control] * 100). Assays were performed in at least triplicate, and data were compared using unpaired *t* tests.

### Macrophage assays.

J774A.1 macrophages were counted using an automated cytometer (Countess II; Thermo Fisher) and seeded at 2.5 × 10^5^ cells/ml 24 h prior to initiation of the coculture in DMEM plus glutamate plus FBS plus pen/strep. The medium was replaced with DMEM without FBS, phenol red, or pen/strep immediately prior to the experiment. C. albicans strains were subcultured in YPD medium for 4 to 6 h, washed 3 times in phosphate-buffered saline (PBS), counted using the automated cytometer, and added to the macrophages at the indicated multiplicity of infection (MOI). In some experiments, the fungal cells were stained with fluorescein isothiocyanate (FITC) prior to the coculture.

### Germination within macrophages.

FITC-stained C. albicans were coincubated with macrophages in phenol red-free DMEM in 8 chamber slides (Ibidi) at an MOI of 1:1 for 2 h. Subsequently, the medium was aspirated and cocultures were fixed with 2.7% paraformaldehyde, counterstained with calcofluor white (CFW), and maintained in PBS. Bright-field microscopy and fluorescence microscopy were performed with an Olympus IX81 microscope, and germ tube lengths were measured using SlideBook 6 software. Experiments were performed in triplicate, with >30 cells measured per strain. Unpaired Student’s *t* tests were performed using Prism 8.

### Estimation of phagosome acidity.

Phagosomal pH was estimated using LysoTracker Red, as described previously (11). Briefly, C. albicans cells stained with FITC were coincubated (MOI 1:1) with macrophages preloaded with the acidophilic dye Lysotracker Red for 1 h in phenol red-free DMEM. Samples were fixed with 2.7% paraformaldehyde. Projections of Z-stack fluorescent images were generated to quantify the red fluorescent intensity of 20 total pixels (10 pixels per side of the fungal cell) at points immediately external to the phagocytosed C. albicans cells. Values representing the area under the curve (AUC) for 20 pixels per cell are reflective of phagosome acidity. A total of >20 internalized cells were measured per strain. Experiment performed in triplicate. Unpaired Student’s *t* tests were performed for AUC values in Prism 8.

### Macrophage association assay.

Scarlet fluorescent C. albicans cells were pregrown as described above and then coincubated with J774A.1 macrophages stained with NucBlue and ActinGreen under optimal conditions (37°C, 5% CO_2_) in an imaging plate reader (Cytation 5; BioTek). Cocultures were briefly centrifuged to synchronize phagocytosis under each set of conditions. Macrophage associations were automatically calculated over several fields of view by quantifying the number of red C. albicans cells associated with the green macrophages (Gen5 software). NucBlue was used for autofocus purposes. Unpaired Student's *t* tests were used for statistical analysis.

### Phagocytosis assay.

Scarlet fluorescent C. albicans cells were pregrown in alternative carbon sources, coincubated with J774A.1 macrophages stained with nucBlue for 60 min, and then fixed with 2.7% paraformaldehyde and counterstained with (membrane-impermeant) FITC. Several fields of view were imaged using the Cytation 5 plate reader, and phagocytosis rates were calculated automatically by the use of Gen5 software with the following formula: [(total number of red cells) − (number of red fluorescent cells plus number of green fluorescent cells)/(total number of fluorescent red cells)] * 100.

### Fungal survival assay.

Fungal survival during coincubation with macrophages was measured as previously described (74). Macrophages were seeded at 2.5 × 10^5^ cells/ml in a 96-well plate 24 h prior to coincubation. C. albicans strains were added at 1 × 10^5^ cells/ml in wells with or without macrophages, followed by a series of six 1:5 serial dilutions. Microcolonies of C. albicans were counted using an inverted microscope in the wells with a discernible number of colonies. Results are represented as percentages of fungal colonies surviving coculture (number of colonies in the presence of macrophages/number of colonies without macrophages * 100) (*n* = 6). Unpaired Student’s *t* tests were performed in Prism 8.

### Macrophage survival assay.

Macrophage survival was measured via detection of lactate dehydrogenase (LDH) in culture supernatants using the CytoTox96 nonradioactive cytotoxicity assay (Promega). Log-phase C. albicans cells were added at an MOI of 1:1 with macrophages in a 96-well plate and coincubated overnight in phenol red-free DMEM. LDH released by infected macrophages was quantified as described by the manufacturer, corrected for spontaneous LDH release, and compared to the data determined for chemically lysed uninfected macrophages (*n* = 6). Unpaired Student’s *t* tests were performed in Prism 8.

### *In vivo* disseminated candidiasis.

Disseminated C. albicans infection was performed as previously described (23). Cells were grown to mid-log phase overnight, centrifuged, washed, counted, and resuspended in PBS. A total of 10 female 7-to-9-week-old ICR mice per strain were infected via tail vein injection with 5 × 10^6^ cells/ml in 100 μl PBS for a final concentration of 5 × 10^5^ injected cells. Mice were monitored 3 times daily for signs of infection and euthanized when moribund. All animal experiments were conducted according to protocols approved by the Animal Welfare Committee of the University of Texas Health Science Center at Houston. The Mantel-Cox test was performed to statistically compare survival curves.
