# Supplementary material for: Multiple Alternative Carbon Pathways Combine To Promote Candida albicans Stress Resistance, Immune Interactions, and Virulence
Source: mBio. 2020 Jan 14;11(1):e03070-19. doi: 10.1128/mBio.03070-19 (PMC6960290; doi:10.1128/mBio.03070-19)
Supplement: FIG S1 [file mBio.03070-19-sf001.pdf]

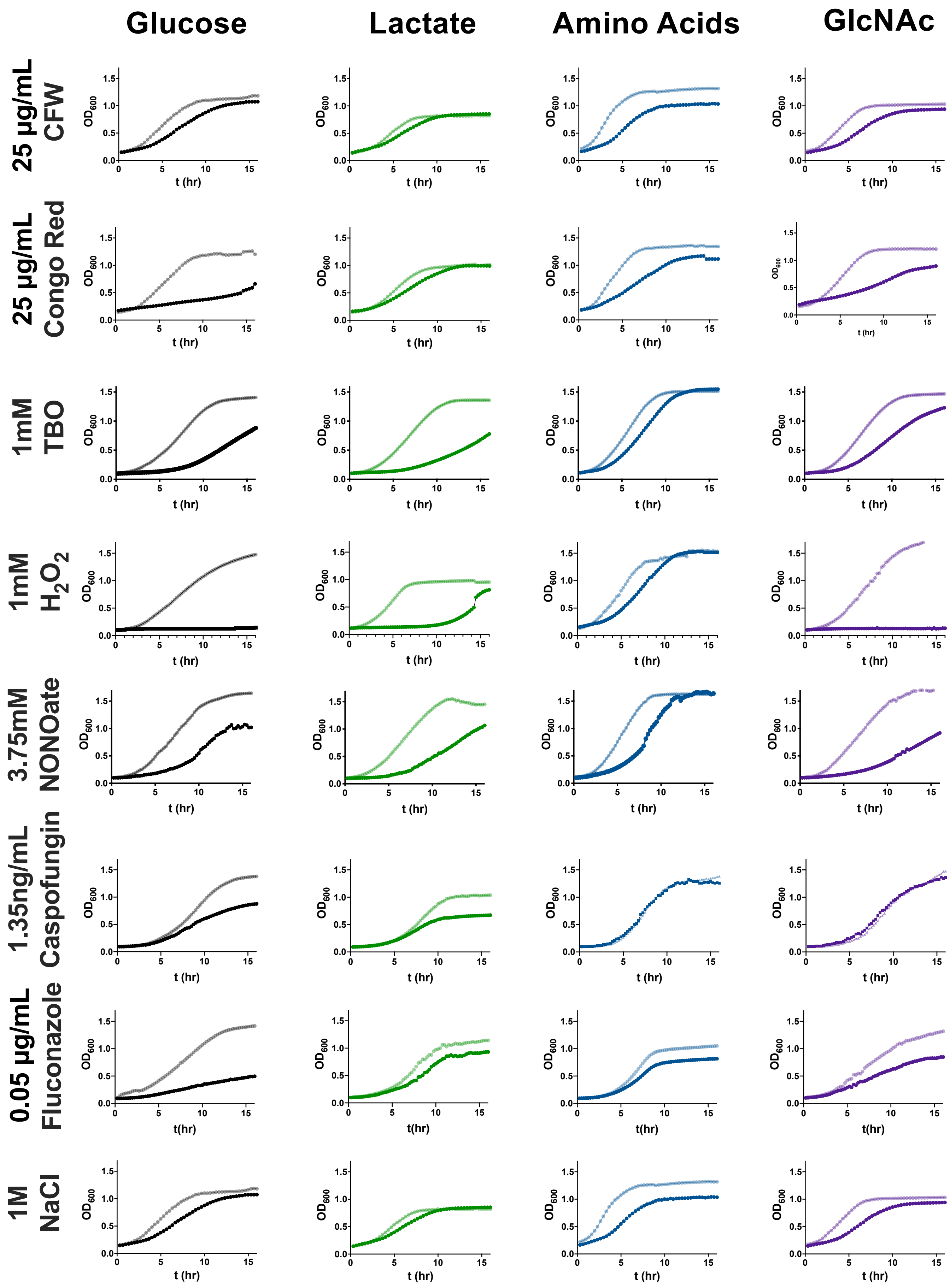

Glucose
Lactate
Amino Acids
GlcNAc
25 µg/mL CFW
25 µg/mL Congo Red
1mM TBO
1mM H_2O_2
3.75mM NONOate
1.35ng/mL Caspofungin
0.05 µg/mL Fluconazole
1M NaCl
OD_{600}
t (hr)
