# Supplementary figures and images for: Multiple Alternative Carbon Pathways Combine To Promote Candida albicans Stress Resistance, Immune Interactions, and Virulence
Source: mBio. 2020 Jan 14;11(1):e03070-19. doi: 10.1128/mBio.03070-19 (PMC6960290; doi:10.1128/mBio.03070-19)

## 10mM TBO

A

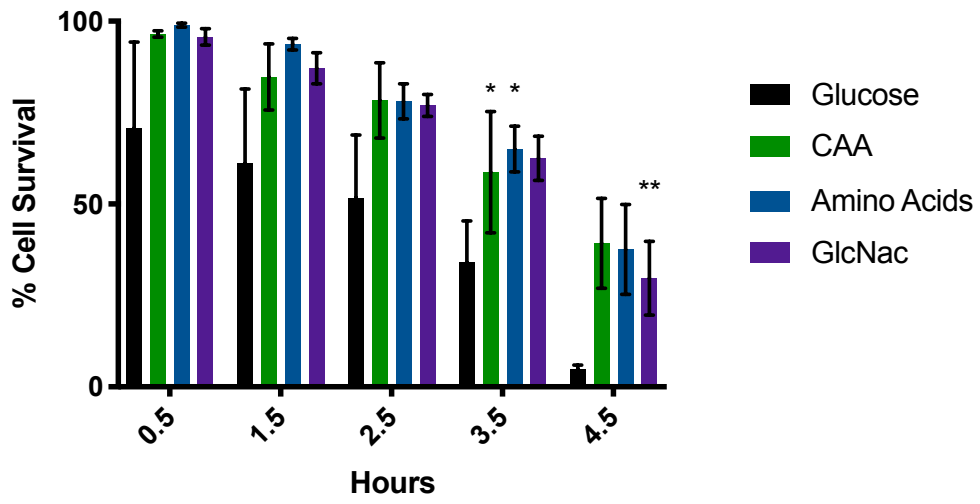

B

## 2M NaCl

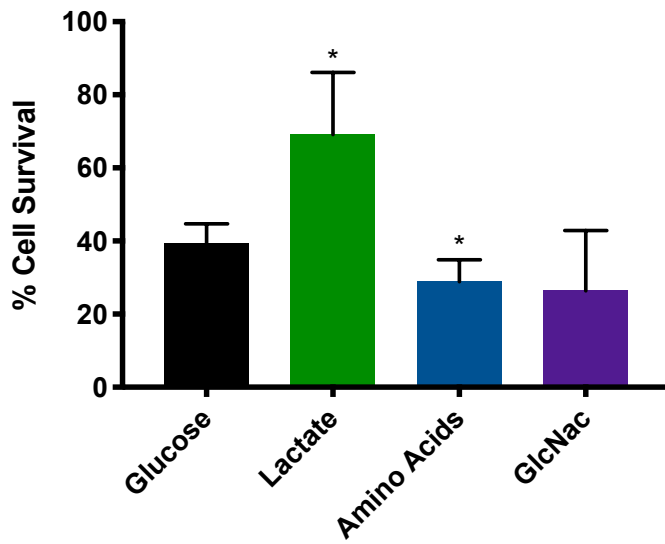

Supplement: FIG S2 [file mBio.03070-19-sf002.pdf]

## Phagosome Acidity

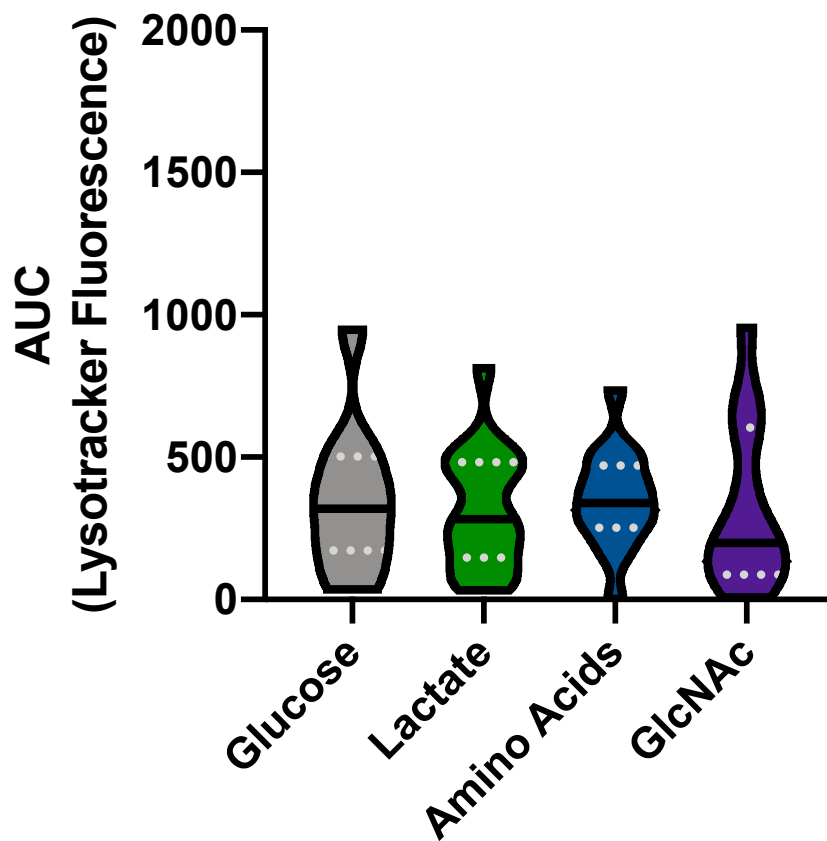

Supplement: FIG S3 [file mBio.03070-19-sf003.pdf]

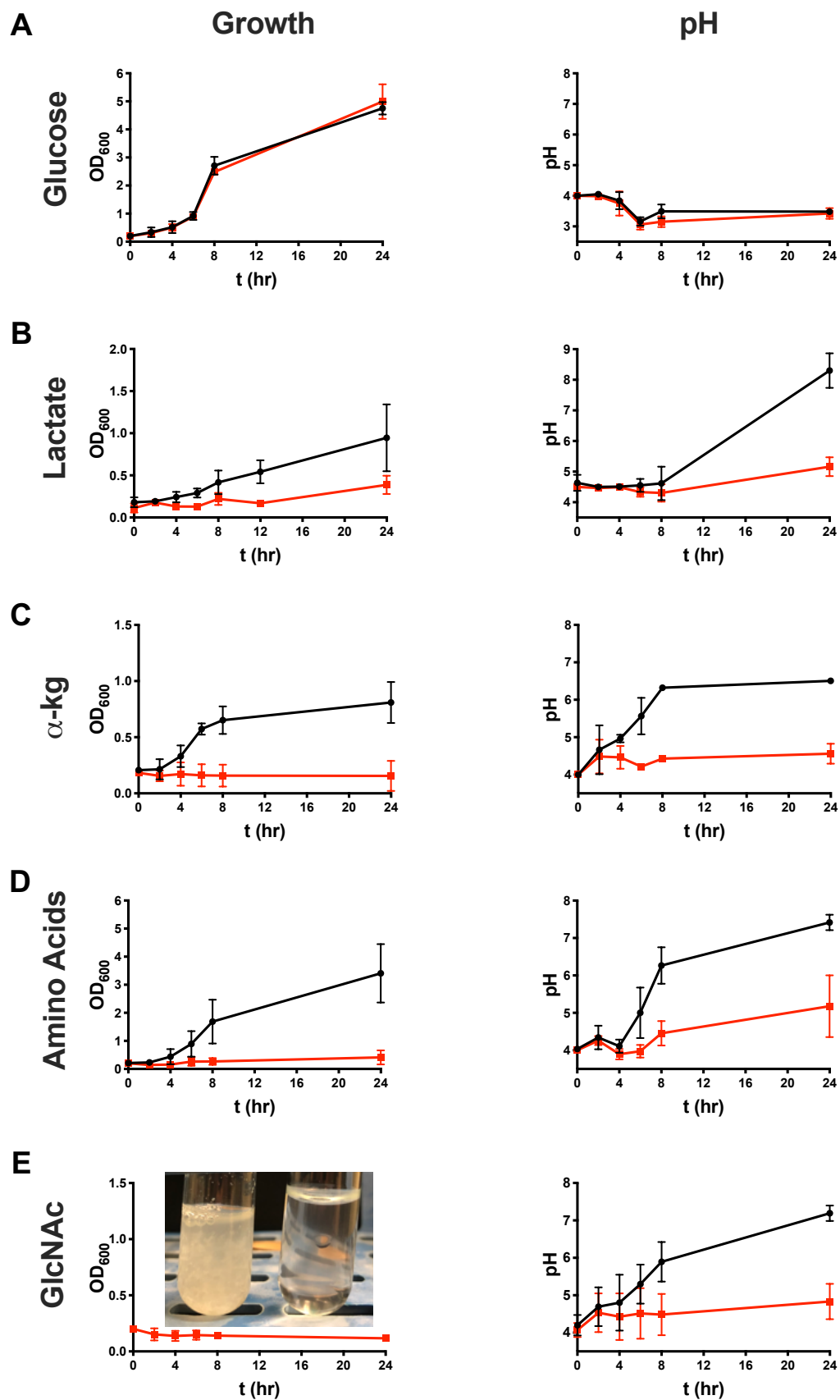

Supplement: FIG S4 [file mBio.03070-19-sf004.pdf]

**SC5314**

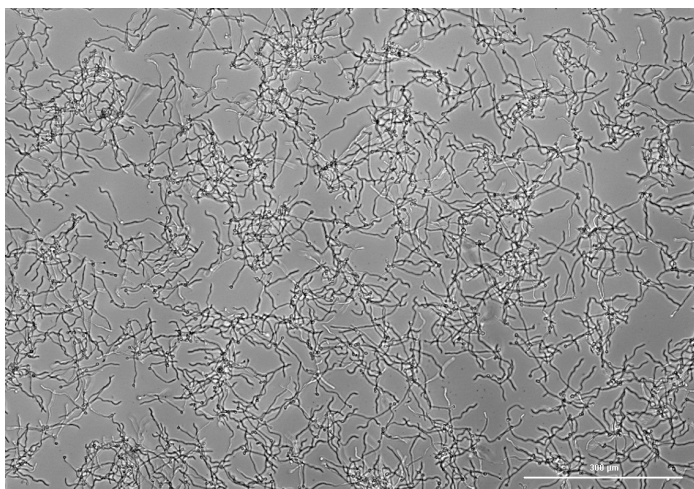

ΔΔΔΔ

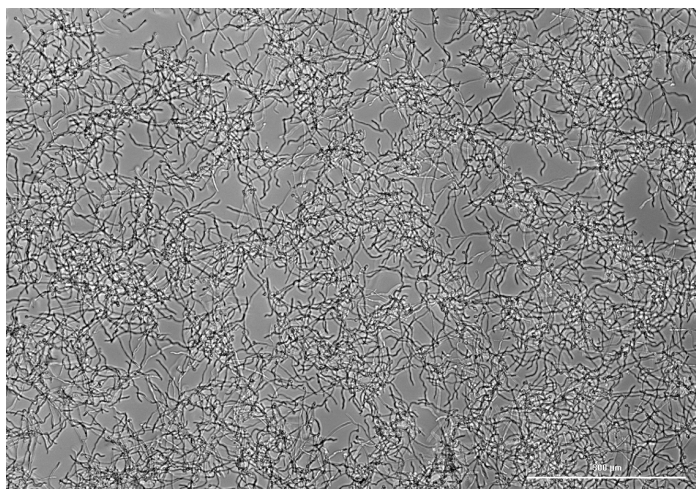

Supplement: FIG S5 [file mBio.03070-19-sf005.pdf]

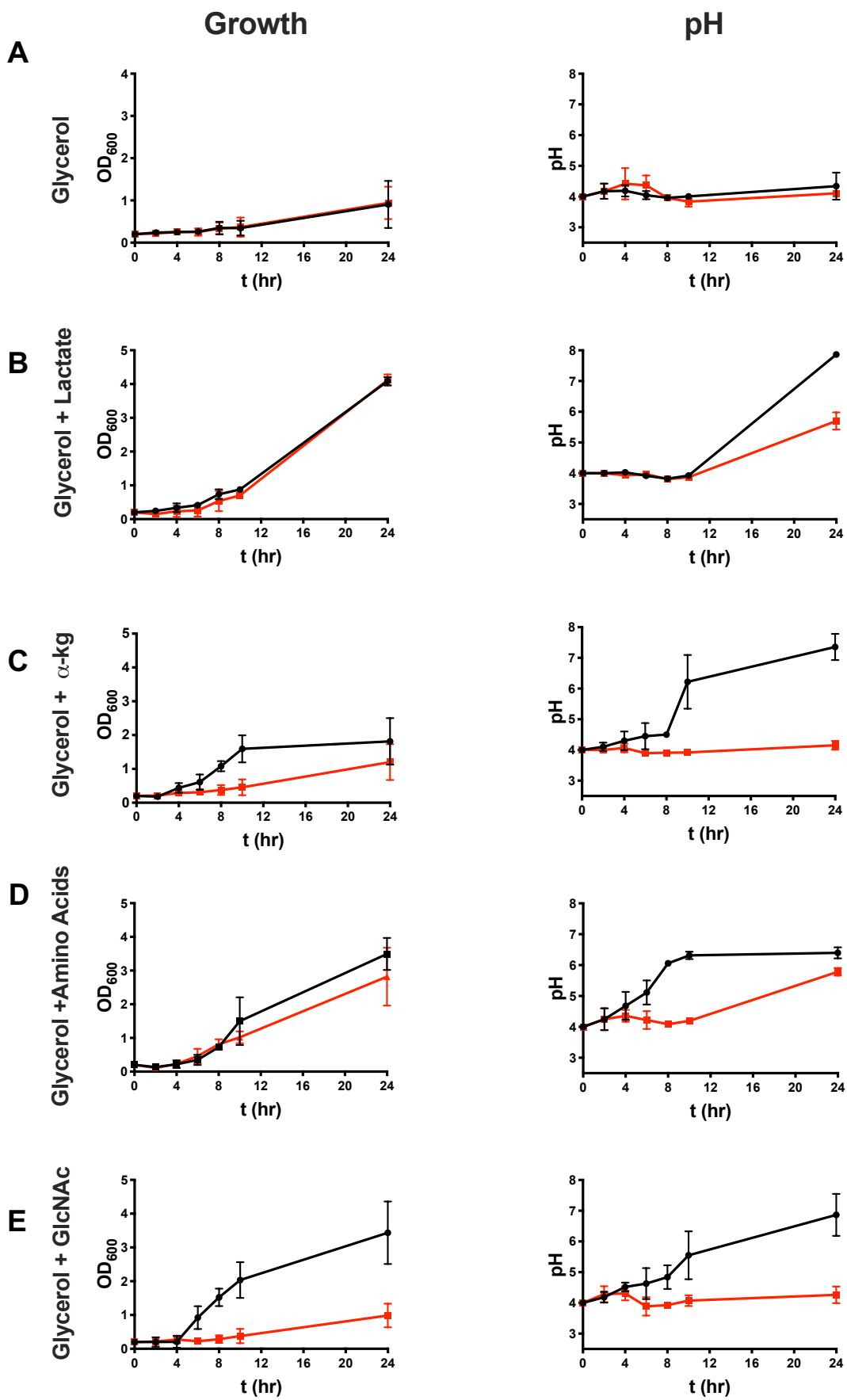

Supplement: FIG S6 [file mBio.03070-19-sf006.pdf]
